# Use of hormones and risk of venous thromboembolism

**DOI:** 10.61622/rbgo/2024FPS02

**Published:** 2024-04-02

**Authors:** Venina Isabel Poço Viana Leme de Barros, André Luiz Malavasi Longo de Oliveira, Denis Jose do Nascimento, Eduardo Zlotnik, Marcelo Melzer Teruchkin, Marcos Arêas Marques, Paulo Francisco Ramos Margarido

**Affiliations:** 1 Clínica Obstétrica Hospital das Clínicas Universidade de São Paulo São Paulo SP Brasil Clínica Obstétrica, Hospital das Clínicas, Universidade de São Paulo, São Paulo, SP, Brasil.; 2 Clínica Obstétrica Hospital das Clínicas Universidade de São Paulo São Paulo SP Brasil Clínica Obstétrica, Hospital das Clínicas, Universidade de São Paulo, São Paulo, SP, Brasil.; 3 Departamento de Obstetrícia e Ginecologia Faculdade de Medicina Universidade Federal do Paraná Curitiba PR Brasil Departamento de Obstetrícia e Ginecologia, Faculdade de Medicina, Universidade Federal do Paraná, Curitiba, PR, Brasil.; 4 Hospital Israelita Albert Einstein São Paulo SP Brasil Hospital Israelita Albert Einstein, São Paulo, SP, Brasil.; 5 Hospital Moinhos de Vento Porto Alegre RS Brasil Hospital Moinhos de Vento, Porto Alegre, RS, Brasil.; 6 Universidade do Estado do Rio de Janeiro Rio de Janeiro RJ Brasil Universidade do Estado do Rio de Janeiro, Rio de Janeiro, RJ, Brasil.; 7 Universidade Federal do Estado do Rio de Janeiro Rio de Janeiro RJ Brasil Universidade Federal do Estado do Rio de Janeiro, Rio de Janeiro, RJ, Brasil.; 8 Hospital Universitário Universidade de São Paulo São Paulo SP Brasil Hospital Universitário, Universidade de São Paulo, São Paulo, SP, Brasil.

## Abstract

•The risk of venous thromboembolism (VTE) is not increased in women using long-acting reversible contraceptive methods (LARCs) with progestogens.

•Oral contraceptives with levonorgestrel or norgestimate confer half the risk of VTE compared to oral contraceptives containing desogestrel, gestodene or drospirenone.

•Progestogen-only pills do not confer an increased risk of VTE.

•Women using transdermal contraceptive patches and combined oral contraceptives (COCs) are at an approximately eight times greater risk of VTE than non-users of hormonal contraceptives (HCs), corresponding to 9.7 events per 10,000 women/years.

•Vaginal rings increase the risk of VTE by 6.5 times compared to not using HC, corresponding to 7.8 events per 10,000 women/years.

•Several studies have demonstrated an increased risk of VTE in transgender individuals receiving hormone therapy (HT).

•Hormone therapy during menopause increases the risk of VTE by approximately two times, and this risk is increased by obesity, thrombophilia, age over 60 years, surgery and immobilization.

•The route of estrogen administration, the dosage and type of progestogen associated with estrogen may affect the risk of VTE in the climacteric.

•Combined estrogen-progesterone therapy increases the risk of VTE compared to estrogen monotherapy.

•Postmenopausal HT increases the risk of thrombosis at atypical sites.

## Recommendations

- The individual risk of VTE must be carefully assessed before initiating any HT.
- Women at risk of VTE (personal or family history) and/or with a history of cardiovascular disease may benefit from the use of progestogen-only HC without increasing the risk of thromboembolic events.
- Transdermal HT appears to have a reduced risk of VTE compared to the oral route in the transgender population.
- A plausible strategy to mitigate the risk of VTE in risk groups is the concomitant initiation of prophylactic anticoagulation and HT, especially in the initial 6-12 months of treatment.
- Although data on the risk of VTE in transgender women in HT undergoing surgery are not available, we suggest stopping it two to four weeks before major surgery with immobilization.
- Data on risk of VTE in transgender men on testosterone HT are very limited, but current studies suggest this risk is either non-existent or irrelevant.
- Micronized progesterone or dydrogesterone has a lower risk than other progestogens, and this risk is greater during the first year of replacement.
- Transdermal estrogen replacement has shown to be safer than the oral route.

## Background

The use of hormones can occur throughout a woman’s life. From menacme, for the treatment of abnormal uterine bleeding (AUB) to contraception and, finally, the climacteric. In addition, there is the challenge of hormone use in transgender women. All these situations can increase the risk of venous thromboembolism (VTE).

## Use of hormones and contraception: what are the risks of VTE?

Hormonal contraceptives (HC) pose a varied risk of VTE. Next, the risks of the main HC methods are explained.

## Long-acting reversible contraceptives (LARCs) and risk of VTE: which one is the best option?

The evolution of HC allowed women to have excellent control of their fertility, in addition to benefiting from its extracontraceptive effects. However, there are adverse events and, among them, the feared risk of VTE that remains the most relevant and under continuous evaluation. According to the World Health Organization (WHO), progestogen-only contraceptive methods do not increase the risk of VTE, acute myocardial infarction (AMI) and cerebrovascular accident (CVA).^(1)^ The LARCs available in Brazil are the etonorgestrel-releasing implant (ENG) and the levonorgestrel-releasing intrauterine system (LNG-IUS). The low failure rates of LARCs are mainly because these methods are independent of users’ motivation to be effective.^(2)^ Among the factors interfering with the use of HCs, the concern with VTE has been intensely investigated and, since 1998, the WHO established that users of oral HC containing estrogen and progestogen are three to six times more likely to have thromboembolic events. Thus, LARCs constitute the best option for women stratified as being at high risk for VTE.^(3)^

## What is the risk of VTE with long-lasting HCs?

Reviews that analyzed the use of progestogen-only HCs did not show an increase in the risk of thrombogenic effect. The thrombogenic effect of progestogen-only HCs on the risk of thromboembolic events did not show a statistically significant difference that could be considered in contraceptive counseling, and these may be prescribed for women with a history of deep vein thrombosis and/or thrombophilia.^(4)^

## What is the risk of cardiovascular events with the use of LARCs?

Arterial thrombosis is represented by AMI and CVA, rare diseases in menacme, although also associated with combined hormonal methods. On the other hand, hormonal LARCs containing progestogens are not associated with an increased risk of AMI and CVA and may be prescribed even for women with these diseases.^(4,5)^ According to the WHO, in women at risk for venous or arterial thrombosis, or even with the occurrence of some of these events in the past, both the ENG implant and the LNG-IUS can be used.^(1)^

## When can LARCs be inserted?

Long-acting reversible contraceptives can be inserted at any time of the menstrual cycle. It is not mandatory that the woman is having her period for insertion, if it is certain that she is not pregnant. When in doubt, a pregnancy test should be performed. The best approach for inserting the LNG-IUS or ENG implant is to wait for the next menstrual cycle. In women with heavy flow, the LNG-IUS should be inserted at the end of menstruation, with the flow greatly reduced or outside the menstrual period, excluding the possibility of pregnancy.^(1)^

## Are there other benefits of these methods besides not increasing the risk of VTE?

Long-acting reversible contraceptives and in particular, the LNG-IUS have shown effectiveness in the treatment of AUB with a reduction in menstrual flow and improvement in hemoglobin and ferritin levels. The lowest dose of levonorgestrel acts directly on the uterus, inhibiting the endometrial synthesis of estrogen receptors and making the endometrium insensitive to circulating estradiol, therefore having an antiproliferative effect leading to its atrophy.^(5,6)^ Long acting reversible contraceptions (LARCs) and in particular, etonogestrel (ENG) implants are also considered the most suitable methods for women in vulnerable conditions, such as homeless people, drug users, sex workers, among others, given their easy insertion and for not depending on the intrauterine location. This vulnerable group is at greater risk of unwanted pregnancies, multiparity, unsafe abortion, prematurity, fetal death, postpartum depression, suicide attempts, violence, sexual infections, and other complications.^(7)^ Levonorgestrel-releasing intrauterine system and ENG implants may be indicated for breastfeeding women, including insertions immediately after childbirth, as they are not related to thromboembolic events, although expulsion rates are higher in the case of the LNG-IUS. They can be inserted six to eight weeks after delivery to reduce the incidence of expulsion. Long-acting reversible contraceptives with progestogen do not alter milk production and the infant’s growth and development.^(7)^

## What is the risk of VTE with the use of combined oral contraceptives (COCs)?

**Figure 1:**
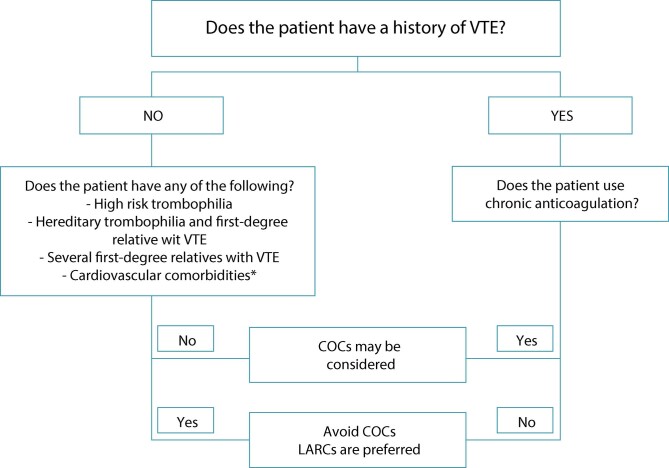
Flowchart of recommendations for the use of contraceptives and risk of venous thromboembolism VTE: venous thromboembolism; COCs: combined oral contraceptives; LARCs: long-acting reversible contraceptives

Source: Adapted from Abou-Ismail et al. (2020).^(14)^

Combined oral contraceptives are widely used as a contraception method, and also bring benefits such as in the treatment of dysmenorrhea, AUB, acne and premenstrual tension. However, as they do increase the risk of VTE, clinicians should perform a clinical evaluation before prescribing them. All COC formulations increase the risk of VTE, but this risk can be minimized by choosing formulations with less thrombogenic hormones, such as levonorgestrel or estetrol for example, or even by reducing the amount of estrogen, such as lowering from 50 to 35 mcg of ethinyl estradiol (EE).^(9)^ In this sense, there is a known interaction between different progestogens and EE. Progestogens with less androgenic action (gestodene, desogestrel and norgestimate) and those with antiandrogenic action (cyproterone acetate and drospirenone) have a greater associated risk of VTE than compounds with progestogens with greater androgenic effect (levonorgestrel, norethisterone, norethindrone and dienogest).^(8,9)^ Also, the change in the estrogenic component, such as the use of estradiol and estetrol confers a lower thrombogenic potential to the COC.^(10)^ The risk of VTE among women who are not users, not pregnant and not taking hormones is of 1-5/10,000 woman-years. Among oral contraception users, the risk of VTE is 3-9/10,000 woman-years. It is important to point out, as a parameter, that the risk is still much higher during pregnancy, of 5-20/10,000 woman-years, and in the postpartum period, of 40-65/10,000 woman-years.^(11)^ The incidence increases with age; in women aged 30-34 years, it is 2.5/10,000 woman-years, and in women aged 60-64 years, it is 9.3/10,000 woman-years.^(12)^ Estrogens increase the serum concentration of factors of coagulation, such as prothrombin, factors VII, VIII and X and fibrinogen, and reduce that of anticoagulants, such as proteins C and S and antithrombin. This risk is greater in women with hereditary thrombophilia. However, the investigation of thrombophilia should be restricted to patients with a personal or family history of VTE, since most women with hereditary thrombophilia will not have VTE, just as most women with VTE will not have hereditary thrombophilia.^(13)^ The increased risk of VTE is greater in the first six months and then decreases up to the 12^th^ month of use. After the first year, the risk stabilizes close to the risk of non-users.^(13)^ In the case of surgery planning in which COC suspension is indicated, in order to avoid increasing the risk already inherent to the surgical procedure, the COC should be suspended in the previous month, the period necessary to reduce the risk of VTE. After surgery, wait three months for the reintroduction of COC to mitigate the risk of VTE in the postoperative period. Figure 1 summarizes the main indications of different HC methods and the risk of VTE.

## What are the risks of VTE in the transgender population?

The terms “transgender” and “gender incongruence” describe a situation where an individual’s gender identity differs from their external sexual anatomy at birth.

The objectives of gender affirmation in transgender women are the suppression, as far as possible, of masculine characteristics and the safe induction of feminine characteristics. Hormone therapy can be performed in several ways, affirmation surgeries, in addition to other procedures, such as waxing or speech therapy.^(15,16)^ Epidemiological data suggest that 0.3% to 0.6% of the adult population is transgender (there are about 25,000,000 transgender people worldwide), but the actual prevalence depends on the definition used to classify a person as transgender.^(17)^ For example, in studies that include only individuals who have undergone HT or gender-affirming surgery, the reported prevalence was 7-9 per 100,000 people.^(18)^ In studies including the transgender status based on self-report, the prevalence was approximately 871 per 100,000 people.^(19)^ Patients who want to affirm the their gender identity must be aware of the risks and benefits of HT and/or surgery, and must be able to give their consent.^(20,21)^

## What are the goals of HT in the transgender population?

The usual goal of HT in the transgender population is to induce physical changes to match gender identity, keeping hormone levels in the normal physiological range for the target sex.^(22)^

## What types of estrogens are used in HT for gender affirmation?

Estrogens are basically divided into two categories: natural human estrogens (17β-estradiol [E2], estrone [E1] and estriol [E3]) and non-human derivatives (derived from the urine of pregnant mares [conjugated equine estrogens – CEEs] or esterified from vegetable sources).^(23)^

The dose used, usually higher than that used for hormone replacement therapy (HRT) in women, will depend on the desired physical changes, the type of estrogen and the route of administration.^(21)^

## What are the precautions for transgender women?

There should be a careful assessment of HT self-medication, both past and current, and the risk-benefit of transgender women undergoing treatment.^(24)^ Several studies have demonstrated an increased risk of VTE in transgender individuals who receive cross-sex HT, particularly in transgender women using estrogen. Most of the information is extrapolated from data and clinical studies carried out to evaluate contraception and climacteric HT.^(25)^

However, this extrapolation is limited, as there are large differences in doses used and the duration of use.

## What are the short-term complications of HT?

The most frequent short-term complications of HT are cardiovascular events, including VTE, and their prevalence is related to the type and doses used, and especially to the route of administration. The oral route induces the hepatic first-pass effect, with an increase in prothrombotic factors, while the non-oral route, particularly the transdermal route, does not seem to induce an increase in VTE.^(25)^ This may be determinant in the choice of initial HT, in which the transdermal route becomes the preferred one in transgender women with a personal history of spontaneous VTE, a family history of VTE, or carriers of thrombophilia. It is important to remember that HT is not elective for this population, but an absolute necessity to obtain the desired phenotype, and in many places, transgender women are on the margins of society, not having access to qualified professionals for their prescription.^(25)^ As a consequence, estrogens are often obtained illegally and used as self-medication without guidance regarding composition, doses and safe routes, especially for the specific risk group for VTE.

Another point for reflection is that non-oral routes of HT are usually more expensive than the oral route hence, inaccessible to most people.^(26)^ A plausible strategy to mitigate the risk of VTE in risk groups is the concomitant initiation of prophylactic anticoagulation with HT, especially in the initial 6-12 months of treatment.^(26)^ Several studies have reported that CEEs, which are more used in the United States, are more thrombogenic than E2, which is more used in Europe.^(25)^ In a retrospective study of more than 1,000 transgender individuals, the incidence of venous thromboembolic complications ranged from 2% to 6% in transgender women treated with oral EE.^(27,28)^ This rate was approximately 20 times higher than that of the male control population. In a follow-up study of the same cohort, no increased risk was observed in users of estrogen preparations other than EE, and based on these observations, current guidelines do not recommend the use of EE.^(29)^ However, VTE has also been observed with other estrogen formulations. In a study of 214 transgender women who received transdermal or oral estradiol, or estradiol gel, 11 (5.1%) had VTE. No events were observed in control groups of cisgender men or women. Other associated risk factors in those who experienced VTE included immobilization after surgery, smoking, or a hypercoagulability disorder.^(30)^ In an electronic medical record-based cohort study including 2,842 transgender women matched with approximately 48,000 cisgender men and 48,000 cisgender women, transgender women had a higher incidence of VTE than both control groups.^(31)^ Most transgender women were receiving oral estradiol at a mean daily dose of 4 mg (range 1-10 mg), the same as those who did not have VTE. The difference was more pronounced in the follow-up of patients, with two- and eight-year absolute risk differences of 4.1 and 16.7, respectively, per 1,000 people, for cisgender men, and 3.4 and 13.7, respectively, per 1,000 people for cisgender women. This pattern is different from that observed in postmenopausal women on HT, in which the risk of VTE is higher in the first year of use and then progressively decreases. This suggests that long-term monitoring is critical in this population.

Although data on the risk of VTE in transgender women on HT undergoing surgery are not available, we suggest stopping estrogen therapy two to four weeks before major surgery with immobilization. Once these individuals are fully recovered and return to their usual activities, estrogen HT can be resumed, usually within four weeks.^(31)^ As the prevalence of thrombophilia appears to be the same in the transgender population and in the general population, routine pre-HT screening is not suggested.^(15)^

## What are the precautions regarding androgen suppression therapy?

Most HT regimens for gender affirmation in transgender women include a second drug with the aim of suppressing the production or counteracting the effects of androgens, particularly testosterone.^(23)^ The most used drug for this purpose is spironolactone, a potassium-sparing diuretic that interacts with hormone steroid receptors, especially androgen receptors, by inhibiting the production of androgens and 5α-reductase, the enzyme that converts testosterone into dihydrotestosterone (DHT).^(23)^ Other drugs used for this purpose are 5α-reductase inhibitors (finasteride), androgen receptor blockers (flutamide), progestogens and gonadotropin secretion agonist/antagonist hormones. There is no association between these drugs and an increased incidence of VTE.

## Is there a higher risk of VTE in HT for transgender men?

Data on the risk of VTE with testosterone HT for transgender men are very limited, but current studies suggest this risk is non-existent or irrelevant.^(25)^

## Hormone replacement therapy in the climacteric: what is the risk of VTE?

Hormone therapy in the climacteric may or may not increase the risk of VTE. Next, the main recommendations are discussed.

## How to assess the risk of thrombosis in HT during the climacteric?

**Figure 2:**
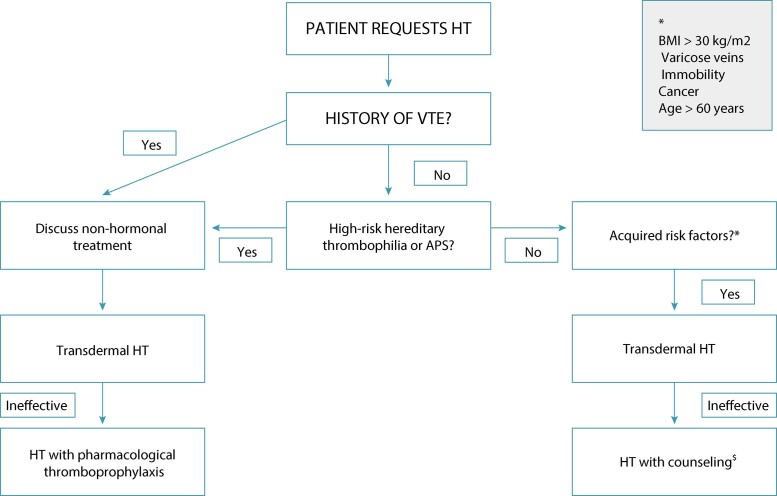
Flowchart for deciding on hormonal treatment or not in the climacteric and risk of venous thromboembolism. *The main acquired risk factors are described. There may be others. ^$^Counseling means being alert to the signs of VTE risk, as well as avoiding risky situations such as immobilization, dehydration and planning surgeries. HT: hormone therapy; VTE: venous thromboembolism; APS: antiphospholipid syndrome; BMI: body mass index Source: Adapted from Lekovic et al. (2017).^(32)^

The recommendations for HT in the climacteric and the risk of VTE are displayed in figure 2.

## Is there an increased risk of VTE with HT?

Beral et al.^(33)^ summarized the results of classic studies of HT and VTE risk. These were prospective, placebo-controlled studies of more than 20,000 women during an observation period of four to nine years. In summary, they stated that HT significantly increases the risk of VTE, with a relative risk (RR) of 2.16. The ESTHER study comprised 155 cases with HT and 381 controls.^(34)^ The results showed not only a significant RR for VTE in users of oral estrogen replacement therapy (RR: 3.5; confidence interval [CI]: 1.8- 6.8]) compared to women without treatment, but also in women undergoing transdermal treatment (RR: 4; CI: 1.9-8.3]).^(34)^ However, a recent study of 80,396 women in the United Kingdom^(35)^ did not demonstrate an increase in the risk of VTE with transdermal hormone replacement, and a Swedish study of more than 1 million patients demonstrated the safety of this route.^(36)^ A study with tibolone in postmenopausal women showed an increased risk of cerebrovascular accident (RR: 2.2) but not of VTE in these patients.^(37)^ A broad review of HRT with testosterone in the climacteric for hypoactive sexual desire was recently published.^(38)^ The risk of VTE with the use of transdermal testosterone by monitoring free testosterone within reference values without other acquired factors seems to be minimal, although the results of the studies are controversial.^(39)^

## Patient using combined contraceptives presents an episode of VTE. How to proceed?

The patient will be anticoagulated for a minimum period of 3-6 months and the LARC insertion should be planned before the end of anticoagulation. This way, the patient is not left unprotected against unwanted pregnancy.^(40)^

## Final considerations

Long-acting reversible contraceptives are highly effective methods and associated with high rates of continuity and satisfaction when the woman is properly advised. They have a strong impact on female health indicators without increasing the risk of VTE, unlike combined HC. Although COCs are a widely used contraceptive method, they must be prescribed with care. The gynecologist must be able to understand the risk situations, minimize them, and indicate the best method for the woman. Hormone therapy continues to be a very important tool in the treatment of the climacteric. The ability to choose the ideal HT preparation for each patient must be based on an understanding of the various clinical and metabolic effects of HT depending on composition, dosage and mode of application. The transdermal route for HT posed the lowest risk for VTE. Several studies have demonstrated an increased risk of VTE in transgender individuals receiving cross-sex HT, particularly in transgender women using estrogens. However, most of the information is extrapolated from data and clinical studies conducted to evaluate contraception and climacteric HT.

## References

[1] World Health Organization, Johns Hopkins Bloomberg School of Public Health, Center for Communication Programs (2018). Family planning: a global handbook for providers: evidence-based guidance developed through worldwide collaboration.

[2] Trussell J (2011). Contraceptive failure in the United States. Contraception.

[3] van Hylckama Vlieg A, Hermerhorst FM, Vandenbroucke JP, Doggen CJ, Rosendall FR (2009). The venous thrombotic risk of oral contraceptives, effects of oestrogen dose and progestogen type: results of the MEGA case-control study. BMJ.

[4] Tepper NK, Whiteman MK, Marchbanks PA, James AH, Curtis KM (2016). Progestin- only contraceptive and thromboembolism: a systematic review. Contraception.

[5] Kaunitz AM, Bissonnette F, Monteiro I, Lukkari-Lax E, Muysers C, Jensen JT (2010). Levonorgestrel- releasing intrauterine system or medroxyprogesterone for heavy menstrual bleeding: a randomized controlled trial. Obstet Gynecol.

[6] Sakamoto LC, Malavasi AL, Karasin AL, Frajzinger RC, Araújo MR, Gebrim LH (2015). Prevenção de gestações não planejadas com implante subdérmico em mulheres da Cracolândia, São Paulo. Reprod Clim.

[7] Federação Brasileira das Associações de Ginecologia e Obstetrícia (2021). Métodos anticoncepcionais reversíveis de longa duração.

[8] Morimont L, Haguet H, Dogné JM, Gaspard U, Douxfils J (2021). Combined oral contraceptives and venous thromboembolism: review and perspective to mitigate the risk. Front Endocrinol.

[9] Grandi G, Facchinetti F, Bitzer J (2022). Confirmation of the safety of combined oral contraceptives containing oestradiol on the risk of venous thromboembolism. Eur J Contracept Reprod Health Care.

[10] Dinger JC, Heinemann LA, Kühl-Habich D (2007). The safety of a drospirenone containing oral contraceptive: final results from the European Active Surveillance Study on oral contraceptives based on 142,475 women-years of observation. Contraception.

[11] Heinemann LA, Dinger JC, Assmann A, Minh TD (2010). Use of oral contraceptives containing gestodene and risk of venous thromboembolism: outlook 10 years after the third generation "pill scare". Contraception.

[12] Nascimento CM, Machado AM, Guerra JC, Zlotnik E, Campêlo DH, Kauffman P (2019). Consenso sobre a investigação de trombofilia em mulheres e manejo clínico. Einstein (São Paulo).

[13] Bloemenkamp KW, Rosendaal FR, Helmerhorst FM, Vandenbroucke JP (2000). Higher risk of venous thrombosis during early use of oral contraceptives in women with inherited clotting defects. Arch Intern Med.

[14] Abou-Ismail MY, Citla Sridhar D, Nayak L (2020). Estrogen and thrombosis: a bench to bedside review. Thromb Res.

[15] Safer JD, Tangpricha V (2019). Care of the transgender patient. Ann Intern Med.

[16] Conron KJ, Scott G, Stowell GS, Landers SJ (2012). Transgender health in Massachusetts: results from a household probability sample of adults. Am J Public Health.

[17] Reisner SL, Conron KJ, Tardiff LA, Jarvi S, Gordon AR, Austin SB (2014). Monitoring the health of transgender and other gender minority populations: validity of natal sex and gender identity survey items in a U.S. national cohort of young adults. BMC Public Health.

[18] Herman JL, Flores AR, O'Neill KK (2022). How many adults and youth identify as transgender in the United States?.

[19] Collin L, Reisner SL, Tangpricha V, Goodman M (2016). Prevalence of transgender depends on the "case" definition: a systematic review. J Sex Med.

[20] Winter S, Diamond M, Green J, Karasic D, Reed T, Whittle S (2016). Transgender people: health at the margins of society. Lancet.

[21] Gooren LJ (2011). Clinical practice. Care of transsexual persons. N Engl J Med.

[22] Weinand JD, Safer JD (2015). Hormone therapy in transgender adults is safe with provider supervision; a review of hormone therapy sequelae for transgender individuals. J Clin Transl Endocrinol.

[23] Gooren L (2005). Hormone treatment of the adult transsexual patient. Horm Res.

[24] Randolph JF (2018). Gender-affirming hormone therapy for transgenders female. Clin Obstet Gynecol.

[25] Becerra Fernández A, Luis Román DA, Piédrola Maroto G (1999). Morbidity in transsexual patients with cross-gender hormone self-treatment. Med Clin (Barc).

[26] Mullins ES, Geer R, Metcalf M, Piccola J, Lane A, Conard LA (2021). Thrombosis risk in transgender adolescents receiving gender-affirming hormone therapy. Pediatrics.

[27] Asscheman H, T'Sjoen G, Lemaire A, Mas M, Meriggiola MC, Mueller A (2014). Venous thrombo-embolism as a complication of cross-sex hormone treatment of male-to-female transsexual subjects: a review. Andrologia.

[28] Asscheman H, Giltay EJ, Megens JA, de Ronde WP, van Trotsenburg MA, Gooren LJ (2011). A long-term follow-up study of mortality in transsexuals receiving treatment with cross-sex hormones. Eur J Endocrinol.

[29] Wierckx K, Elaut E, Declercq E, Heylens G, De Cuypere G, Taes Y (2013). Prevalence of cardiovascular disease and cancer during cross-sex hormone therapy in a large cohort of trans persons: a case-control study. Eur J Endocrinol.

[30] Getahun D, Nash R, Flanders WD, Baird TC, Becerra-Culqui TA, Cromwell L (2018). Cross-sex hormones and acute cardiovascular events in transgender persons: a cohort study. Ann Intern Med.

[31] Wierckx K, Mueller S, Weyers S, Van Caenegem E, Roef G, Heylens G (2012). Long-term evaluation of cross-sex hormone treatment in transsexual persons. J Sex Med.

[32] Lekovic D, Miljic P, Dmitrovic A, Thachil J (2017). How do you decide on hormone replacement therapy in women with risk of venous thromboembolism?. Blood Rev.

[33] Beral V, Banks E, Reeves G (2002). Evidence from randomized trials on the long-term effects of hormone replacement therapy. Lancet.

[34] Canonico M, Orger E, Plu-Bureau G, Conard J, Meyer G, Lévesque H (2007). Hormone therapy and venous thromboembolism among postmenopausal women: impact of the route of estrogen administration and progestogens: the ESTHER study. Circulation.

[35] Vinogradova Y, Coupland C, Hippisley-Cox J (2019). Use of hormone replacement therapy and risk of venous thromboembolism: nested case-control studies using the QResearch and CPRD databases. BMJ.

[36] Sundell M, Spetz Holm AC, Fredrikson M, Hammar M, Hoffmann M, Brynhildsen J (2022). Pulmonary embolism in menopausal hormone therapy: a population-based register study. Climacteric.

[37] Cummings SR, Ettinger B, Delmas PD, Kenemans P, Stathopoulos V, Verweij P (2008). The effects of tibolone in older postmenopausal women. N Engl J Med.

[38] Uloko M, Rahman F, Puri LI, Rubin RS (2022). The clinical management of testosterone replacement therapy in postmenopausal women with hypoactive sexual desire disorder: a review. Int J Impot Res.

[39] Nicholson M, Chan N, Bhagirath V, Ginsberg J (2020). Prevention of venous thromboembolism in 2020 and beyond. J Clin Med.

[40] James AH (2017). Pregnancy, contraception and venous thromboembolism (deep vein thrombosis and pulmonary embolism). Vasc Med.

